# 

**Published:** 1918-10-12

**Authors:** 


### Passing Events and Topics.

### Doctor's Estates.

DR. C. E. GLASCOTT, of Budleigh Salterton, for thirty-five years hon. surgeon to the Royal Eye Hospital, Manchester, and afterwards one of the vice-presidents, has left £18,621, and Dr. H. B. Robinson, of Upper Wimpole Street, W., senior surgeon to St. Thomas's Hospital, surgeon to the East London Hospital for Children, and examiner in surgery for the London and Manchester Universities, has left £11,195.

### Public Health Problems.

A SIXTH course of lectures and discussions on "Public Health Problems under War and After-War Conditions" will be held in the Lecture Hall of the Institute of Public Health, 37 Russell Square, W.C., on Wednesdays in October, November, and December, at 4 P.M. The first lecture, on "Anthropometry and National Health," was given on October 9 by Professor Arthur Keith, M.D. The coming October lectures include one on "Racial Reconstruction and the Proposed Ministry of Health," by C. W. Saleeby, Esq., M.D.; "The Obviation of Ship-borne Infections," by W. M. Willoughby, Esq., M.D.; "The Prevention and Abortive Treatment of Venereal Disease," by Lt.-Col. L. W. Harrison, M.B., when Sir George H. Makins, G.C.M.G., will be in the chair. Other lecturers include Professor J. M. Beattie, M.D., on "Infection and Disinfection in War-time," when Col. J. G. Adami, M.D., will be in the chair; Miss Jane Walker, M.D., on "The Tuberculous Soldier"; and Sir John Collie, M.D., on "The Care of Pensioners and Disabled Combatants in Relation to National Health and Wealth."

### Matrons' and Sisters' Appointments.

Municipal Isolation Hospital, South Shields.—Mrs. S. C. Cowan has been appointed matron. She was trained at the Royal Infirmary, Edinburgh, and at the Belvidere Fever Hospital, at which latter institution she has since been ward sister, night superintendent, and home sister.

Royal Victoria Hospital, Dover.—Miss Gertrude Vergette has been appointed matron. She was trained at St. Bartholomew's Hospital, London, where she has since been sister of the massage department.

### Forthcoming Events.

British Hospitals Association.—Conference, Friday, October 18, at 3 p.m. at St. Thomas' Hospital, "The Voluntary Hospitals and the Proposed Ministry of Health Bill."

The College of Nursing.—Royal Infirmary, Carlisle, Friday, October 11, at 5.30 p.m. County Hospital, Lincoln, on Monday, October 14, at 3.30 p.m. All nurses in the vicinity are cordially invited to attend these meetings, which will be addressed by the Organising Secretary of the College.

The Royal Sanitary Institute, 90 Buckingham Palace Road, S.W. 1.—Friday, October 18, at 2 p.m., visit to Messrs. Pilkington's Tile and Pottery Company's Factory, Clifton Junction. 6.30 p.m., a discussion at the College of Technology, Manchester, on "Welfare Work in Factories and Workshops," to be opened by Professor J. Radcliffe, M.Sc.Tech. (Member of Council), and Miss Ethel Brown (Welfare Secretary, Messrs. John Bright and Brothers, Rochdale). Chairman, Philip Boobbyer, M.D., M.S. (M.O.H. Nottingham) (Member of Council).

